# Semi-Quantitative Assessment of Regional Cerebral Blood Flow Changes Following Extracranial–Intracranial Bypass Using SPECT: A Single-Center Retrospective Study

**DOI:** 10.3390/jcm15155981

**Published:** 2026-07-31

**Authors:** Ying-Chia Wu, Yen-Chih Fong, Meng-Hsiun Tsai, Yuang-Seng Tsuei, Chung-Hsin Lee

**Affiliations:** 1 Department of Neurosurgery, Neurological Institute, Taichung Veterans General Hospital, Taichung 40705, Taiwan; g312985@gmail.com; 2 Department of Management Information Systems, National Chung Hsing University, Taichung 40227, Taiwan; johnsonfongwade03@gmail.com (Y.-C.F.); mht@nchu.edu.tw (M.-H.T.); 3 College of Medicine, National Chung Hsing University, Taichung 40227, Taiwan; astrocytoma2001@yahoo.com.tw; 4 Department of Surgical Medicine, Chung Shan Medical University, Taichung 40201, Taiwan; 5 Department of Neurosurgery, Taichung Veterans General Hospital, Taichung 40705, Taiwan; 6 Institute of Medicine, Chung Shan Medical University, Taichung 40201, Taiwan

**Keywords:** extracranial–intracranial bypass, STA-MCA anastomosis, moyamoya disease, cerebral blood flow, SPECT, hemodynamic assessment, steno-occlusive disease

## Abstract

**Background/Objectives:** Extracranial–intracranial (EC-IC) bypass, particularly superficial temporal artery to middle cerebral artery (STA-MCA) anastomosis, is used to augment cerebral perfusion in selected patients with moyamoya disease or atherosclerotic steno-occlusive disease. This study evaluated the impact of STA-MCA bypass on regional cerebral perfusion using a semi-quantitative SPECT area-based method. **Methods:** Of 40 patients screened between June 2018 and June 2022, 15 with complete paired pre- and postoperative CTA and SPECT data were included. Postoperative imaging was performed at a median of 12 months (range, 6–15 months). Proportional perfusion area in the basal ganglia and temporal regions was calculated using Labelme annotation, Canny edge detection, and Green’s theorem, normalized to total brain area. Paired comparisons and exploratory subgroup analyses were performed. Normality was assessed by Shapiro–Wilk test; Wilcoxon signed-rank tests served as sensitivity analyses. **Results:** Mean age was 56 ± 15.7 years (40% female). All 16 anastomoses were patent. Postoperative perfusion area increased significantly in the basal ganglia (mean difference 0.24, 95% CI 0.13–0.35, *p* = 0.0003) and temporal regions (0.34, 95% CI 0.08–0.60, *p* = 0.012). Wilcoxon tests confirmed these findings (basal ganglia *p* = 0.0003; temporal *p* = 0.008). In unilateral cases, ipsilateral and contralateral basal ganglia improvements did not differ significantly (*p* = 0.839). Results should be interpreted as exploratory given the limited sample size. **Conclusions:** STA-MCA bypass was associated with significant increases in semi-quantitative perfusion area on SPECT, particularly in the basal ganglia. The lack of ipsilateral–contralateral difference is consistent with a possible global hemodynamic effect, but this interpretation remains hypothesis-generating. The pragmatic SPECT method is feasible for perioperative monitoring yet requires further validation. Larger prospective studies with standardized protocols are warranted.

## 1. Introduction

Extracranial–intracranial (EC-IC) arterial bypass has been utilized to treat various intracranial pathologies, including moyamoya disease, arterial stenosis or occlusion, complex aneurysms not amenable to endovascular treatment, and secondary cerebral ischemia [1,2]. In patients with carotid stenosis or occlusion, bypass improves cerebral blood flow (CBF) at rest and augments cerebrovascular reserve during periods of increased metabolic demand [2,3,4]. Consequently, it can reduce further ischemic events, reverse the vascular steal phenomenon, and potentially enhance long-term neurocognitive function in appropriately selected patients [5].

Because EC-IC bypass may reduce stroke risk, restore cortical thickness, and improve cognitive outcomes, objective pre- and postoperative hemodynamic monitoring is essential to confirm revascularization success [6,7]. The objective of this study was to investigate the impact of STA-MCA bypass on regional cerebral perfusion using single-photon emission computed tomography (SPECT) as a semi-quantitative surrogate marker, with particular focus on the basal ganglia and temporal regions—areas critical for motor, cognitive, and executive functions [8,9]. We further examined whether surgical laterality influenced the pattern of perfusion improvement (ipsilateral vs. contralateral regions).

## 2. Materials and Methods

### 2.1. Patients and Surgical Procedure

During the study period from June 2018 to June 2022, 40 patients who underwent STA-MCA bypass at our institution were screened for the availability of pre- and postoperative brain computed tomography angiography (CTA) and rCBF-SPECT studies. After excluding patients with incomplete pre- or postoperative SPECT and/or CTA imaging or loss to follow-up, 15 patients with complete paired imaging data were included in the final analysis. Preoperative and postoperative clinical evaluations, including neurological examination and symptom assessment, were performed by the attending neurosurgical team. This introduces a potential source of observer bias, as the same team that performed the surgery also conducted the clinical assessments. Postoperative CTA for anastomosis patency assessment and rCBF-SPECT for hemodynamic evaluation were performed at a median of 12 months after surgery (range, 6–15 months). One patient was imaged at 6 months, one at 15 months, and the remaining 13 patients at 12 months. The timing was individualized according to clinical status and logistical considerations; no rigid standardized imaging protocol was employed. Although the majority of patients were assessed at a consistent 12-month interval, the presence of two outliers and the absence of a rigid protocol are acknowledged as limitations, as intervening clinical events could theoretically influence imaging findings.

Inclusion criteria were: (1) patients with intracranial steno-occlusive disease or moyamoya disease who received either unilateral or bilateral superficial temporal artery to middle cerebral artery (STA-MCA) EC-IC bypass; (2) availability of preoperative CTA and at least one postoperative CTA; and (3) availability of preoperative rCBF-SPECT and at least one postoperative rCBF-SPECT study.

One patient underwent bilateral bypass surgery, resulting in a total of 16 anastomoses across 15 patients. This patient was included in the overall pre–post perfusion analyses. For the ipsilateral versus contralateral subgroup analysis, only the 14 patients with unilateral bypass were analyzed to permit unambiguous classification of laterality.

### 2.2. Image Processing and rCBF Measurement

**Figure 1:**
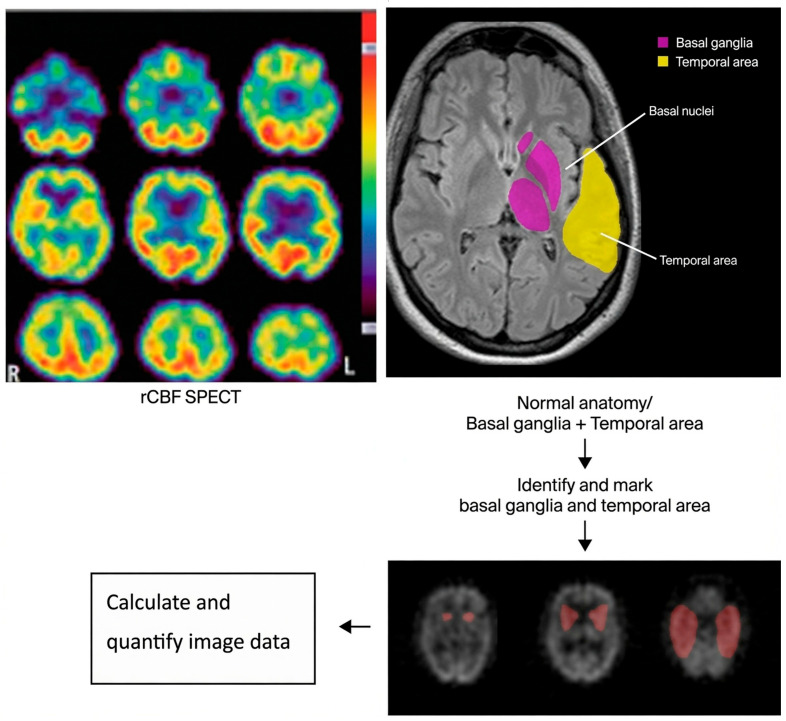
Detailed flowchart of the semi-quantitative rCBF area measurement pipeline using Labelme annotation, Canny edge detection, and Green’s theorem. The process includes slice selection, polygonal annotation with Labelme, binary mask generation, contour detection, area calculation, and normalization.

We focused on the basal ganglia and temporal regions for perfusion assessment, as these areas are crucial for motor control, cognition, and executive function and are commonly affected in steno-occlusive cerebrovascular disease [6,8,9]. The detailed image processing pipeline is illustrated in Figure 1 and consists of the following steps:

- Slice Selection: Preoperative and postoperative SPECT images were co-registered with corresponding CTA when available. Two independent observers (one neurosurgeon and one neuroradiologist) selected the most representative axial slice at the level of the basal ganglia (approximately at the anterior commissure–posterior commissure line) and the temporal lobes, ensuring clear visualization of the lenticulostriate territories and Sylvian fissure.
- Region of Interest (ROI) Annotation using Labelme: Selected DICOM images were converted to 8-bit grayscale PNG format and imported into Labelme (version 5.0.1). Polygonal annotations were manually drawn to delineate areas of detectable tracer uptake.
- Labelme Annotation Protocol:
  - The “Create Polygons” tool (shortcut: Ctrl + N) was used to trace the boundaries of the bilateral basal ganglia (caudate nucleus, putamen, and globus pallidus) and temporal lobes (superior, middle, and inferior temporal gyri).
  - Annotations were performed in a blinded manner with respect to pre- or postoperative status to minimize bias.
  - For each region, label names were assigned as BG_Left, BG_Right, Temp_Left, and Temp_Right.
  - Practical techniques included using the mouse wheel for precise zooming, holding the space bar to pan the image, using Ctrl + Z to undo, and right-clicking to close the polygon. For regions with fuzzy boundaries (common in SPECT), a conservative 1–2 pixel outward adjustment was applied, followed by manual vertex editing. Newer versions of Labelme with Segment Anything Model 2 (SAM2) integration were optionally used for initial automatic segmentation, followed by manual refinement to ensure anatomical accuracy.
- Binary Mask Generation and Edge Detection: Exported JSON files were parsed using custom Python scripts (Python 3.9 with OpenCV 4.5). Contours were extracted using the Canny edge detection algorithm (low threshold = 50, high threshold = 150, aperture size = 3).
- Area Calculation: The area enclosed by each closed contour was computed using Green’s theorem (shoelace formula), providing sub-pixel accuracy for irregular anatomical regions.
  $$A=\frac{1}{2}\;\left|\sum_{i=1}^{n-1}(x_{i}y_{i+1}-x_{i+1}y_{i})+(x_{n}y_{1}-x_{1}y_{n})\right|$$
- Normalization: The ROI area was normalized by dividing it by the total brain area (excluding ventricles and extracerebral spaces) on the same axial slice, yielding a proportional perfusion area metric (unitless ratio).
- Reliability Assessment: Intra-rater and inter-rater reliability were evaluated on 20 randomly selected images (10 preoperative and 10 postoperative) by two independent observers using a two-way random-effects model for absolute agreement, single measures [ICC(2,1)]. ICC values exceeded 0.88 for both basal ganglia and temporal regions. Ninety-five percent confidence intervals for the ICC were not calculated in the original analysis and are therefore unavailable; this is acknowledged as a limitation, and the high point estimates should be interpreted with appropriate caution. Custom Python scripts are available from the corresponding author upon reasonable request.

This pragmatic semi-quantitative method offers a practical, anatomically specific approach for clinical hemodynamic assessment without requiring absolute quantification. However, it remains two-dimensional and operator-dependent, and future studies should validate this approach against fully quantitative techniques such as PET or arterial spin labeling MRI.

### 2.3. Surgical Intervention and Follow-Up

All patients underwent direct STA-MCA bypass via end-to-side anastomosis of the parietal branch of the superficial temporal artery to a cortical M4 branch of the middle cerebral artery. Standard postoperative follow-up included clinical evaluation, CTA assessment of anastomosis patency, and rCBF-SPECT for hemodynamic assessment. All imaging was independently evaluated by two experienced neuroradiologists or neurosurgeons.

### 2.4. Statistical Analysis

Categorical variables are presented as counts and percentages. Continuous variables are expressed as mean ± standard deviation (SD). Paired pre- and postoperative comparisons were performed using the paired *t*-test, with mean differences (MD) and 95% confidence intervals (CI) reported. Normality of the pre–post differences was assessed using the Shapiro–Wilk test. Both basal ganglia (*p* = 0.28) and temporal region (*p* = 0.49) differences satisfied the normality assumption. As a sensitivity analysis, Wilcoxon signed-rank tests were also performed and yielded consistent results (basal ganglia *p* = 0.0003; temporal *p* = 0.008). Subgroup analyses were conducted according to surgical laterality and, for unilateral cases, according to ipsilateral versus contralateral regions. These subgroup analyses were considered exploratory and hypothesis-generating; therefore, no formal correction for multiple comparisons was applied. Post hoc power calculations, if mentioned, are for descriptive purposes only and should not be interpreted as evidence of robustness given the small sample size. A *p*-value < 0.05 was considered statistically significant. Analyses were performed using MedCalc for Windows (version 22.032).

## 3. Results

Fifteen patients were included. The mean age was 56 ± 15.7 years, and 40% (*n* = 6) were female. Surgical indications were moyamoya disease (*n* = 3, 20%) and arterial stenosis or occlusion (*n* = 12, 80%). Fourteen patients underwent unilateral STA-MCA bypass, and one patient received bilateral bypasses, resulting in a total of 16 anastomoses. All anastomoses were patent intraoperatively and remained patent on postoperative CTA. One patient experienced a recurrent ischemic stroke in the left temporal cortex nine months after left-sided bypass surgery; no other major perioperative complications were observed (Table 1).

**Figure 2:**
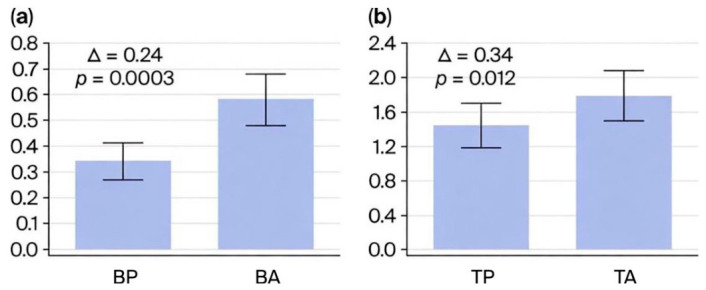
Bar plots showing pre- and postoperative proportions of perfusion area in (**a**) basal ganglia and (**b**) temporal regions; Δ = mean difference; *p*-values from paired *t*-tests.

Quantitative analysis of proportional perfusion area on rCBF-SPECT demonstrated statistically significant postoperative improvement in both regions of interest. For the basal ganglia, the mean difference (MD) was +0.24 (95% CI 0.13–0.35, *p* = 0.0003). For the temporal regions, the MD was +0.34 (95% CI 0.08–0.60, *p* = 0.012). Normality assumptions were met, and Wilcoxon signed-rank sensitivity analyses confirmed the findings (basal ganglia *p* = 0.0003; temporal *p* = 0.008) (Figure 2).

In unilateral cases (n = 14), there was no statistically significant difference in improvement of basal ganglia perfusion between ipsilateral and contralateral sides (overall ipsilateral Δ +0.128 vs. contralateral Δ +0.130, *p* = 0.839) (Table 2). Similar directional improvements were observed in the temporal regions (Left: ipsilateral +0.202 vs. contralateral +0.266, *p* = 0.429; Right: ipsilateral +0.118 vs. contralateral +0.125, *p* = 0.907; Overall: ipsilateral +0.160 vs. contralateral +0.195, *p* = 0.447), although the magnitude was smaller and more variable than in the basal ganglia. These findings are consistent with the hypothesis of a more global hemodynamic effect rather than a strictly localized phenomenon; however, the study was not powered for formal equivalence testing, and this interpretation remains hypothesis-generating.

## 4. Discussion

Low-flow EC-IC arterial bypass, particularly superficial temporal artery to middle cerebral artery (STA-MCA) anastomosis, remains an important revascularization strategy for carefully selected patients with moyamoya disease or atherosclerotic steno-occlusive disease who exhibit impaired cerebrovascular reserve [1,2,10,11]. In the present study, we achieved a 100% postoperative patency rate across 16 anastomoses, which is comparable to or exceeds the 95–99% patency rates reported in contemporary high-volume cerebrovascular centers [12,13].

The core hemodynamic finding of this study—statistically significant postoperative expansion of the proportional perfusion area on semi-quantitative SPECT, with particularly robust and consistent gains in the basal ganglia—aligns well with multiple prior investigations employing various perfusion imaging modalities, including SPECT, CT perfusion, and arterial spin labeling MRI [14,15,16]. Anatomically, direct anastomosis to a cortical M4 branch primarily augments the distal MCA territory. However, the pronounced improvement observed in the basal ganglia, which are predominantly supplied by the lenticulostriate perforators arising from the M1 segment, is noteworthy. This phenomenon likely results from a combination of mechanisms: (1) the overall increase in hemispheric inflow leading to enhanced pressure gradients across perforators, (2) the reversal of the vascular steal phenomenon, (3) the recruitment of leptomeningeal and pial collaterals, and (4) the restoration of cerebral autoregulation capacity [17,18].

Consistent with Mesiwala et al. [2] and Li et al. [4], our semi-quantitative SPECT analysis revealed directional improvement in 13 of 15 patients in the basal ganglia region. Furthermore, Ha et al. [19] reported that increases in normalized cerebral blood flow on arterial spin labeling MRI correlated with the degree of postoperative neovascularization in pediatric moyamoya disease, supporting the notion that perfusion imaging can serve as a surrogate marker of revascularization success. 

In our subgroup analysis (Table 2), improvement in basal ganglia perfusion was similar between patients undergoing left-sided and right-sided surgery. More importantly, in unilateral cases, there was no statistically significant difference between ipsilateral and contralateral basal ganglia improvement (*p* = 0.839). This finding is consistent with the hypothesis that the hemodynamic benefit of STA-MCA bypass may extend beyond the immediate surgical territory [19,20]. Importantly, the absence of a statistically significant ipsilateral–contralateral difference does not constitute formal proof of equivalence, as the study was not powered for equivalence testing. These results should therefore be viewed as supportive of a hypothesis rather than definitive evidence of a global mechanism.

The basal ganglia play a pivotal role in cognitive gating, executive function, motor control, and emotional regulation through their complex cortico-striato-thalamo-cortical loops [6,21]. While the observed perfusion augmentation in this deep gray matter structure is of potential clinical interest, the present study did not include formal neuropsychological testing (e.g., MoCA or detailed cognitive batteries). Therefore, any discussion of potential cognitive implications remains purely speculative and should not be over-interpreted. Prior literature has suggested possible cognitive benefits after EC-IC bypass in selected patients [6,11,22,23,24]; confirmation requires prospective studies with objective neuropsychological assessment.

Although the landmark Carotid Occlusion Surgery Study (COSS) demonstrated no overall benefit of EC-IC bypass over best medical management in patients with symptomatic unilateral internal carotid artery occlusion and misery perfusion [3], subsequent subgroup analyses and selective case series suggest that highly selected patients with recurrent ischemic events despite optimal medical therapy and objective evidence of severe hemodynamic impairment may still derive benefit [25,26,27]. Our real-world Asian cohort adds data supporting individualized application of bypass surgery [28].

Methodologically, the custom semi-quantitative area-proportion method offers practical advantages in daily clinical settings. However, several important limitations must be acknowledged. First, the analysis relies on a single representative axial slice rather than full volumetric data, which may introduce slice-selection bias and loss of three-dimensional information. Second, manual polygonal annotation introduces operator-dependent variability; although mitigated by blinded assessment and high ICC point estimates (>0.88), 95% confidence intervals for ICC were not calculated and residual subjectivity cannot be eliminated. Third, the method lacks direct external validation against quantitative standards such as [^15^O]H_2_O-PET or ASL-MRI. Fourth, the study population is heterogeneous (moyamoya 20%, atherosclerotic 80%), limiting generalizability. Fifth, the small sample size (n = 15) limits statistical power for subgroup analyses and precludes formal equivalence testing; findings must be regarded as exploratory and hypothesis-generating. Sixth, clinical assessments were performed by the treating neurosurgical team, introducing potential observer bias. Seventh, the absence of a rigid standardized protocol for the timing of postoperative CTA and SPECT (most patients imaged at 12 months, with single outliers at 6 and 15 months) remains a limitation, as intervening clinical events could theoretically have influenced findings in the minority of cases imaged outside the 12-month window.

Future directions include larger prospective multicenter studies with standardized imaging protocols, quantitative perfusion imaging, comprehensive neuropsychological assessment, and long-term clinical outcome data. Identification of reliable hemodynamic and clinical responders will further refine patient selection criteria in contemporary revascularization practice [29,30].

## 5. Conclusions

In our single-center study, STA-MCA bypass was associated with a significant increase in semi-quantitative perfusion area on SPECT, with consistent benefits observed in the basal ganglia regardless of surgical laterality. In unilateral cases, the absence of a significant ipsilateral–contralateral difference is consistent with the hypothesis that hemodynamic benefit may extend beyond the immediate surgical territory; this interpretation remains hypothesis-generating and requires confirmation. The pragmatic semi-quantitative SPECT method is feasible for perioperative hemodynamic monitoring but requires further validation against quantitative standards. These preliminary findings should be confirmed in larger prospective multicenter studies incorporating standardized imaging protocols, quantitative perfusion imaging, and long-term clinical outcomes.

## Figures and Tables

**Table 1:** Patient demographics, comorbidities, and surgical characteristics (*n* = 15).

Variable	Value
Age, years (mean ± SD)	56 ± 15.7
Sex, *n* (%)	
Male	9 (60)
Female	6 (40)
Comorbidities, *n* (%)	
Hypertension	6 (40)
Diabetes mellitus	9 (60)
Hyperlipidemia	6 (40)
Atrial fibrillation	0 (0)
Coronary artery disease	2 (13.3)
Prior ischemic stroke	13 (86.7)
Prior hemorrhagic stroke	1 (6.7)
Chronic renal failure (eGFR < 60)	0 (0)
Antiplatelet therapy	10 (66.7)
Diagnosis, *n* (%)	
Moyamoya disease	3 (20)
Arterial stenosis/occlusion	12 (80)
Surgical laterality, *n*	
Unilateral	14
Bilateral	1
Total anastomoses	16 (all patent)

**Table 2:** Subgroup analysis of basal ganglia and temporal perfusion area change according to surgical laterality and ipsilateral vs. contralateral regions (unilateral cases, *n* = 14).

Basal Ganglia Area				
Surgery Side	*n*	Basal Ganglia Δ (Ipsilateral)	Basal Ganglia Δ (Contralateral)	*p*-Value (Ipsi vs. Contra)
Left	7	+0.121	+0.111	0.545
Right	7	+0.134	+0.149	0.316
Overall (unilateral)	14	+0.128	+0.130	0.839
Temporal area				
Surgery Side	*n*	Temporal Δ (Ipsilateral)	Temporal Δ (Contralateral)	*p*-Value (Ipsi vs. Contra)
Left	7	+0.202	+0.266	0.429
Right	7	+0.118	+0.125	0.907
Overall (unilateral)	14	+0.160	+0.195	0.447

## Data Availability

The data presented in this study are available on request from the corresponding author. The data are not publicly available due to privacy and ethical restrictions.
